# Evaluating the COVID-19 impacts on the construction and demolition waste management and resource recovery industry: experience from the Australian built environment sector

**DOI:** 10.1007/s10098-022-02412-z

**Published:** 2022-10-01

**Authors:** Salman Shooshtarian, Savindi Caldera, Tayyab Maqsood, Tim Ryley

**Affiliations:** 1 School of Property, Construction and Project Management, RMIT University, Melbourne, Australia; 2 Cities Research Institute, Griffith University, Brisbane, Australia; 3 School of Engineering and Built Environment, Griffith University, Brisbane, Australia

**Keywords:** Pandemic, Government response, Construction and demolition waste, Circular economy, Supply chain, Australia

## Abstract

The COVID-19 crisis has brought unprecedented challenges to many sectors, including the built environment. The construction and demolition (C&D) waste management and recovery industry is an essential service provider to this sector. Like other industries, this industry has been affected by the pandemic in many ways. However, in Australia, this impact has not been thoroughly investigated. This study, therefore, explores COVID-19 impacts on the Australian C&D waste recovery and construction industry as the major waste consumer and generator. To achieve this aim, a literature review and a series of semi-structured interviews were conducted with 27 participants representing five stakeholder groups (government, construction, waste recovery, material supplying and consultancy) across five Australian states. The research findings established that there is a critical need for leveraging digital technologies, developing business contingency plans, creating coalitions between government and industry, and diversifying supply chains to reduce supply chain risks. This study also uncovered a range of targeted responses and recommendations to manage pandemic-induced disruptions and improve the circular economy in the industry. Our findings can immediately assist industrial practitioners and government decision-makers in managing the impacts of COVID-19 on the waste recovery activities in C&D waste and other waste streams.

**Graphical abstract:**

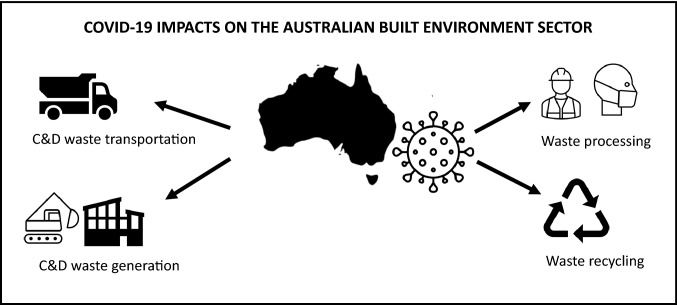

## Introduction

The spread of COVID-19 has been creating significant challenges to many sectors, including housing, building and infrastructure, leading to many alterations and amendments to waste management practices (Alhosani & Liravi 2021; Sharma et al. 2020). In Australia, the first case of the novel coronavirus was reported on 19 January 2020 (Australia Department of Health 2020), and the number of cases has continued to rise ever since. The Australian Government's COVID-19 statistics show that, as of 22 September 2021, Australia had recorded 90,372 total cases. While Australia has been one of the few successful countries in containing the diseases, it was estimated to be one of the most affected economies in the Australasia region. The predictions suggested a shrink of 6.7% in the Australian economy in 2020 (Khadem 2020) and impacting a substantial proportion of small and medium-sized enterprises in construction (Bell et al. 2022). As a result, many industries have faced financial challenges; the waste management and resource recovery industry—waste recovery in short—has not been an exception. The containment of the spread of the COVID-19 pandemic and limitations on commercial activities, mobility, construction and manufacturing sectors have significantly affected waste management operations (Lv et al. 2021). 

While the construction industry is responsible for 44% of the solid waste generated every year in Australia (National Waste Report 2020), the C&D waste recovery industry has been severely affected by this pandemic. Van Fan et al. (2021) maintained that such an impact, in the global context, includes a change in waste amount and composition, frequency and distribution and associated risks. These, in turn, affect waste handling and relevant treatment practices. To reflect and learn from the challenges related to COVID-19, it is critical that both government and industry to re-assess their supply chain risks (Caldera et al. 2022) and determine waste management and resource recovery activities will deliver the most resiliency in the event of another large-scale disruption. Within that context, this study evaluates COVID-19 impacts on the Australian C&D waste recovery and construction industry and provides policy recommendations to further assist the industry with sustainable operation during these challenging times.

### Literature review

In some major Australian states, there has been a significant increase in construction activities to meet a growing population's needs and keep up with economic growth (Australian Bureau of Statistics 2022). Furthermore, Australian state governments are spending public funds to counteract the drop in the economy caused by COVID-19. During the COVID-19 pandemic, the government economic recovery plan proposed new initiatives to boost the construction industry. Among other things, the homebuilder project offered funds to construct a new home, substantially renovate an existing property or purchase a property off-the-plan (Victoria State Revenue Office 2021). This initiative has led to a surge of new developments, particularly residential housing (Yu 2021). The governments also have accelerated investment in government-funded infrastructure projects across Australia (Jotzo et al. 2020).

The management of the ensuing C&D waste has become a major challenge. The construction industry is accountable for 44% of the solid waste generated every year in Australia (National Waste Report 2020). Australia is known to be among the top ten countries listed in the Organisation for Economic Cooperation and Development (OECD) for generating solid waste. In 2018–2019, Australia produced a total of 27 million tonnes of C&D waste (National Waste Report 2020). This emphasises the critical need for a better waste management practice to divert waste from landfills and to improve the resource circular economy (Shooshtarian et al. 2022). Therefore, it is necessary to design and implement a sustainable C&D waste management system due to the increasing amounts of C&D waste, the scarcity of landfills and the long-term effects of C&D waste disposal on the environment, economy and society.

However, to achieve sustainable C&D waste management system the major barriers need to be identified. Several attempts have highlighted these major barriers. To date, the lack of advanced technological capabilities and knowledge (Hyder 2012), the mechanisation of demolition works (Kim 2021), the geographic spread of products to be returned for reuse or recycling (Shooshtarian et al. 2022), inconsistent levy rates across states (Edge Environment Pty Ltd 2012), quality of the recycled material (Udawatta et al. 2015) and attitudes and behaviour towards waste management practices (Kim & Nguyen 2020) have been uncovered as key barriers for C&D waste management. In recent years, COVID-19 adds another layer of complexity to managing the large quantity of C&D waste in Australia, posing additional challenges to waste operators, recyclers and other relevant industry and government decision-makers.

Observations from the industry performance worldwide suggest that industry operators are going through unprecedented difficulties during the pandemic impact (Klemeš et al. 2020; Readling 2020; Waste Advantage 2020). Countries such as the USA have ceased recycling programs in some of their cities, as authorities have become concerned about the risk of COVID-19 spreading in recycling centres (Zambrano-Monserrate et al. 2020).

Some public and private organisations have attempted to discover the impact on the industry by inviting operators to express how their operations have been disrupted. For instance, a survey by the US. National Waste & Recycling Association (2020) discovered that the primary impacts on the industry are employee safety and health concerns, operational best practices, changes in revenues, services, waste streams and waste volumes. The survey also showed that waste hauliers are the most affected operators, staffing accounting for the greatest short-term impact, and two-thirds of participants indicated moderate to severe operations impacts due to COVID-19 restrictions. Another survey, facilitated by Waste Today magazine, showed that around 30% of respondents operating in the US waste management industry had been ordered to close their facilities by state officials or had voluntarily idled one or more facilities (Readling 2020). The survey results specific to the C&D waste stream showed that 50% of inbound flow had been affected from moderately to severely. Other reported impacts included 32% cases of layoffs (a further 21% were considering layoffs), and around 66% of businesses had put one or more purchases on hold. Love and Rieland (2020) state that given worker safety concerns, low market prices for scrap materials, a slowed economy and cheaper alternatives for disposal, many communities and businesses across the US have temporarily suspended the collection of recyclables.

With disruptions in waste recovery activities, the environment is under threat worldwide. There is evidence that demonstrates how this impact is damaging the environment. In some UK rural communities, disrupted services have increased waste mismanagement by 300% (You et al. 2020). Roberts et al. (2020) reported that with fewer options available, traditional waste management practices such as landfills and incineration are replacing more sustainable measures such as recycling, with adverse effects on the environment. Government organisations sometimes exacerbate the issue. For instance, the UK Environment Agency has allowed temporary waste and incineration ash storage at sites that further threaten the environment.

### Research scope, hypothesis and objectives

In Australia, except for a few industry-led inquiries, research on the COVID-19 impacts on waste recovery is limited. One example is the survey facilitated by the Australian Council of Recycling to further understand stakeholders experience during the pandemic (ACOR 2020). ACOR suggested that governments should consider the findings as part of the ongoing response to COVID-19. Also, Waste Management Resource Recovery Association of Australia (WMRR) updates the industry on COVID-19-related policies and measures in different Australian states. In the lack of empirical information demonstrating the effects of the pandemic on the C&D waste recovery business in Australian states, this research, for the first time, attempts to identify the significant implications by collecting the perspectives of key stakeholders.

In consultation with the experts and analysis of relevant literature the following hypothesis was developed:

#### COVID-19 has had negative and uncontrolled impacts on the waste recovery industry across Australia

To test this hypothesis the following objectives were formed:

1. *To identify the impacts of COVID-19 on the C&D waste recovery industry*?
2. *To unveil how has the industry responded to the pandemic-induced challenges?*
3. *To explore the primary adaptive measures to overcome these challenges?*

This timely research will serve to develop environmental policies and practices seeking to mitigate such impacts on waste recovery. The following sections present the research method applied, results from the data collection followed by discussion and conclusions and recommendations.

## Research method

**Fig. 1:**
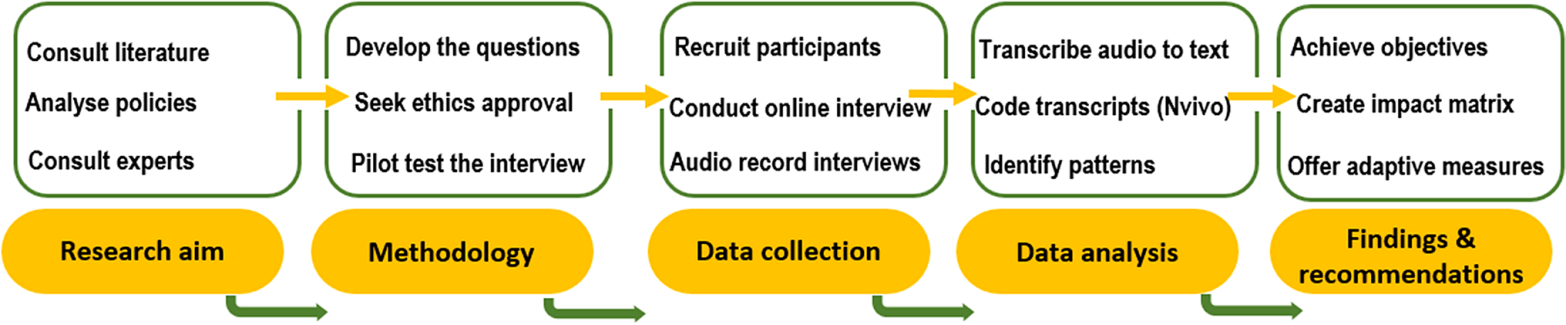
Flowchart of the research process

This study adopted an exploratory approach to understanding the COVID-19 impacts on the Australian C&D waste management and resource recovery industry. This approach enabled the authors to evaluate the available evidence and provide the groundwork for more rigorous studies in the future (Cooper et al. 2006). The authors analysed the background context through existing literature and defined research questions as the preliminary step of the exploratory study as exhibited in Fig. 1.

### Research context

**Fig. 2:**
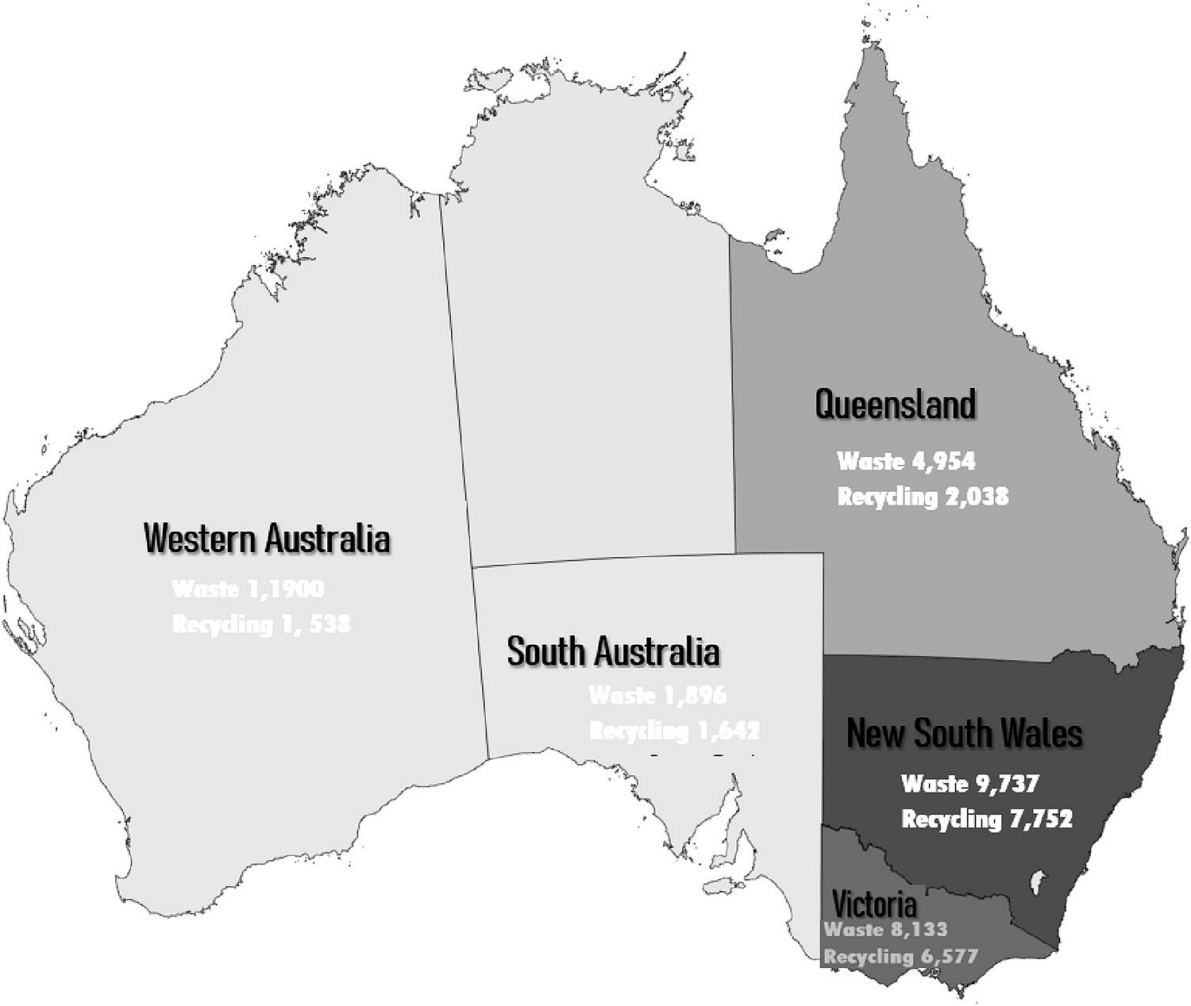
The five study states with associated waste generation and recycling data. Source: National Waste Report (2020)

In Australia, waste recovery activities are regulated at the jurisdictional level. One public organisation in each jurisdiction is responsible for governing waste-related activities (Shooshtarian et al. 2019). Such an organisation also produces information regarding the COVID-19 impact on the industry. This study targeted five major Australian states as a case study to achieve the main research aim. These states are Victoria (Vic), New South Wales (NSW), Queensland (Qld), South Australia (SA) and Western Australia (WA). As illustrated in Fig. 2, these states had the largest rates of waste generation and recovery in 2019.

### Data collection

An online interview method was employed to collect data relevant to the research aim. The interviews were conducted using the Microsoft Teams application. The interview consisted of several questions covering the main issues and opportunities regarding the development of the market for recycled C&D waste materials. As presented in the questions selected and analysed for this study sought participant experiences, their opinion on the impact of COVID-19 on C&D waste recovery activities and issues around the development of the market for recycled C&D waste materials.

To further guarantee the study's internal reliability of interview questions, a pilot interview was conducted with waste-related experts. The feedback from the pilot interview assisted the research team in optimising the interview schedule. Since the study required experts in the field, one of the main selection criteria for interviewees was the adequate experience in dealing with waste management in Australia. According to the literature review conducted in this study and expert advice, the most relevant stakeholder groups for interviews were identified. These groups include those who were involved in developing policy and practice of waste recovery (government officials and construction experts), manufacturing and recycling of construction materials (suppliers and recyclers) and providing consultation services to the other stakeholder groups (consultants).

### Recruitment of research participants

Research participant recruitment was undertaken according to the requirements set by the Australian National Statement on Ethical Conduct in Human Research (National Health and Medical Research Council 2007 (Updated 2018)) and further obligations imposed by the human research ethics committees at RMIT and Griffith universities. The project industry partners, the Australian Sustainable Built Environment National Research Centre (SBEnrc) and the Waste Management and Resource Recovery Association of Australia (WMRR), assisted with the recruitment process. The two organisations provided the contact details of relevant stakeholders from their member networks. WMRR's members consist of waste recycling businesses. SBEnrc members include experts representing industry and government organisations. WMRR is the primary industry stakeholder for this work, while SBEnrc funded the research project. A purposive sampling strategy was deemed the most appropriate approach given the research timeframe. It was employed to recruit a wide range of participants across the C&D waste supply chain representing five Australian jurisdictions.

Email communication was used as the method of recruitment. An email with the project information sheet was sent to a list of participants compiled by the research team in one round. This covered 60 individuals with relevant experience in waste management and the resource recovery sector. The list consisted of the two organisation members and other experts identified by researchers. A reminder email was also sent to those who did not respond in the first round. Interview participation was voluntary, and attending the interview implies informed consent. The investigators maintained the privacy and confidentiality of all interview information as per the human ethics requirements.

### Participant profile

**Fig. 3:**
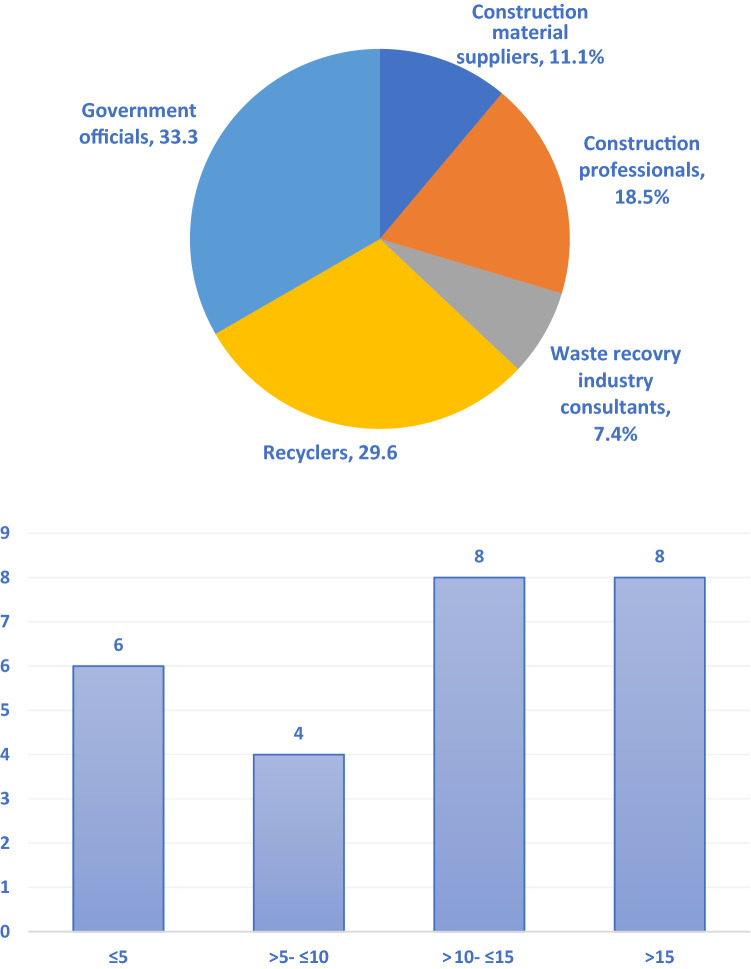
The frequency of stakeholder groups and their experience

The primary stakeholders who have a substantial role in the utilisation of recycled products (e.g. government, recyclers and construction professionals) are well represented in the sample size. As shown in Fig. 3, the largest category type was government officials (9), followed by recyclers (8), professionals working in the construction industry (5), construction material suppliers (3) and waste recovery consultants (2).

The interviewees were based in WA (*n* = 11), Qld (*n* = 7), Vic (*n* = 5), NSW (*n* = 3) and SA (*n* = 1). This shows a wider geographical spread across Australia. In terms of gender, five interviewees were female. The remainder was male, which echoes the gender balance in the construction industry. Furthermore, 20 of interviewees had more than 5 years’ of experience working in C&D waste management space. This suggests that interview responses properly represented the industry's atmosphere during the pandemic.

### Data analysis

Each interview took, on average, 45 min and was audio recorded with prior permission. Audio data were carefully transcribed by a professional transcriber word-for-word. The research team further verified the quality of text data. Analysis of the transcripts was performed using the NVivo Pro 12 application (Di Gregorio 2000). This application facilitates codifying text-based qualitative data. A thematic analysis method was applied to identify emerging themes (Braun & Clarke 2006), with a combination of inductive and deductive reasoning approaches employed. The analysis began with a deductive or theory-driven coding system (a-priori codes) using the literature review/interview guide elements while creating additional new nodes (in vivo codes) inductively from emerging interview data.

## Results

The findings are presented under two themes: (1) COVID-19 impacts on businesses operating in C&D waste and the construction industries and (2) responses to COVID-19 impacts and lessons learned. These two themes are aligned with the first two study’s objectives.

### COVID-19 impacts on businesses and the C&D waste industry

As per findings, the COVID-19 pandemic has created some form of disruption to the C&D waste recovery industry through changes to their working shifts, supply chain interruptions, delays between the construction and demolition cycles, and the inability to retain or recruit a workforce. The interview participants from different industry sectors and government shared their lived experiences in the face of COVID-19 disruptions. The impacts varied across Architecture, Engineering and Construction (AEC) sectors; while public construction projects continued during the outbreak, there were significant impacts on residential and commercial projects. Despite the government economic support packages (e.g. Job Keeper,^1^ the organisation had to reduce employees on some projects. Furthermore, the number of tenders for construction projects was also reduced. An organisation's ability to continue operating on public projects assisted its ability to endure the pandemic situation. Table 1 synthesises the key impacts shared by interview participants across the five states of NSW, QLD, VIC, WA and SA.

**Table 1:** Summary of COVID-19 impacts on businesses and the C&D waste industry

State	Key impacts
NSW	A slight decline in demolition-type activities from C&D materials received by the site
	The decline of waste streams depends on the severity of the state government's reaction
	Review of supplier contracts and rates to align with the new state of the economy
Qld	Supply chain interruptions for material purchased from overseas markets
	Limited material flow and increased effects by the quantum of infrastructure projects that were happening at the same time
	Interruptions to onsite inspections, especially to cultural heritage surveys
	Working remotely and travel restrictions
	Restricted production and inability to export and import as usual
	Increased levels of efficiency in transportation certain operations due to clear roads and transport networks
	The resource recovery sector was impacted by stockpile materials unable to process or get shipping containers to store the materials
Vic	Increasing budgetary constraints
	Dealing with uncooperative industry partners with changed priorities
	The decline in market activities in the AEC industry
	Delays in several projects starting that are considered an economic slump
	Pandemic-induced human resource challenges (reduce the number of employees)
	Reduced tenders for construction projects
WA	
	The waste operation continued as it is considered an essential service
	Self-isolation among the workforce in the operations
	Incentives in the residential market and not commercial have been counteractive to this impact
	Limited resources, both human resources and shortage of materials, particularly steel, timber
	Reduced the number of people working on construction sites. particularly building houses
	Increased pressure to do better, to recycle more C&D material with increasing infrastructure projects
	The residential housing market was doing well, and there has been a positive impact on the broader macroeconomic perspective
	Changes in the priority of the organisation's activities such as bringing forward infrastructure projects commencement dates
SA	The job keeper/job seeker schemes negatively impacted labourers' choosing not to do hard physical work while being paid
	Increase in workload and the labour hour percentage

While many of the interview participants admitted the rapid implementation of COVID-19 safety protocol, including clearly divided shifts, wearing protective equipment and the use of hand sanitisers. Overall, the waste industry addressed many challenges through vertical integration and the continuation of building and construction projects. The following sections describe key impacts with specific interview examples across the five Australian jurisdictions.

#### Interruptions to the regular business operations and the urgent need for safety precautions

Changing of work shifts, interactions with people, logistical arrangements, additional safety precautions and onsite engagements impacted the regular business operations. There was a significant emphasis on rapid interactions and tasks such as prestart checks and cleaning activities. A private sector representative from New South Wales shared, *"COVID affected the interactions with the people. Initially, there was a rush on the masks and the hand sanitisers, but as with all free-market economies, a number of companies re-tasked their production and six weeks later none of us had a problem*" [P2]. Given construction's heavy reliance on onsite and in-person work, COVID-19 lockdowns and social distancing measures have put a great strain on employees within the industry. For example, *"We had a few issues around cultural heritage experts coming up from New South Wales to do cultural heritage surveys. So, certain things become challenging that you can't do virtually. When you're going to do cultural heritage surveys, there are certain experts that are required"* [P5].

A government representative [P8] from Queensland pointed out significant concerns around transport and sanitation. For transport, this related to unknown issues (e.g. tissues, masks) at the time. The government was unable to mobilise and provide clear information immediately. While there was some resource limitation at the beginning (i.e. sanitisers), this was rapidly addressed with hand sanitiser quickly manufactured for industrial use. While paused operations in China impacted the resource recovery sector, there were concerns about having to stockpile materials unable to process or get shipping containers to store the materials.

#### Supply chain interruptions

Most participants experienced some form of supply chain interruptions, especially for material purchased from overseas markets. One government participant shared that this was further exacerbated by the quantum of infrastructure projects that were happening simultaneously. For example, *"It's also the quantum of infrastructure projects that are happening at the same time, so if you think about, you've got Melbourne Metro, Sydney Metro, Cross River Rail (inland rail). There's a lot of major infrastructure projects that are going to be hitting construction around the same time, and that's going to have an impact only on materials*" [P5].

#### Delays between the C&D cycles

Delivering infrastructure projects across the state and delays between the C&D cycles were highlighted as critical impacts. These impacts created higher-order challenges to the organisation in terms of managing the mass balance of C&D required for the sustainable operation of the recycling facility. An interviewee from South Australia [P11] reiterated that these delays impacted the State's logistical and resource recovery operations.

#### Pandemic-induced human resource challenges

While many participants expressed significant operational difficulties, contractors, especially ones with large workforces, encountered several pandemic-induced human resource challenges. These include pay cuts, laying off employees or increasing the workload of existing employees. Three interviewees indicated that their response was to retain the workforce [P5, P6, P11]. However, there was an example of one organisation implementing a 30% pay cut across their business to achieve this goal rather than laying off employees. It was considered a fair approach and enabled them to respond quickly to the impacts. For example, *ʺI think everyone felt quite together; everyone felt quite bonded by it because everyone was doing it together. And the people that performed more, the people that were in the higher income of the stream gave up more… as an executive team, we didn't have to start picking winners and losers. And I think that was incredibly motivating for our team, and I think it was incredibly motivating from our managers that, here is a company saying we want all of us to be in this togetherʺ* [P11]. In this interviewee's experience, Job Keeper and Job Seeker schemes negatively impacted labourers choosing not to do hard physical work while being paid. This phenomenon resulted in a significant increase in workload and the labour hour/cost percentage.

### Responses to COVID-19 impacts and lessons learned

Participants across the five Australian jurisdictions shared their experiences relating to the responses to COVID-19 disruptions and challenges. While most participants described their rapid responses as aligned with emergent safety priorities and efficient management of resources, this experience has also helped them to uncover a range of opportunities. Learning from these COVID realities, participants also developed consciousness around the need to leverage digital technologies, developed business contingency plans, created coalitions between government and industry, diversified supply chains, and reduced supply chain risks. The key responses are as follows:

#### Leveraging digital technologies

The emergent opportunities using real-time data, digital engineering and other online technologies when dealing with such disruptions were highlighted by several participants. For example**, "***I think COVID might have positive repercussions for things like smart cities and real-time data, and digital engineering. I think that those shifts would then support this kind of work".* Another participant engaged in a large transport infrastructure project reiterated the importance of leveraging online technologies (P10). One government sector participant shared their experience in leveraging current digital capabilities of using cloud-based technologies to work with clients and colleagues across different states. **"***I think we'd already moved to Adobe Sign before COVID because we work across three different states. And GIS has been used for modelling, I think we were set up quite well, virtually, prior to COVID, simply because of our expansive breadth"* [P5]***.***

#### Developing contingency plans

The importance of contingency plans within the business continuity planning process was emphasised by two interviewees. For example, one participant shared their experience in developing contingency plans*, ʺOur leadership team identified the potential impacts that COVID was going to have. We put contingency plans in place, response plans in place very early and had communication out to the sites, the ability to work remotely was responded to quite quickly. So as a business, and in terms of potential impacts, we had really good continuity plans in placeʺ* [P1]*.*

#### Creating coalitions between government and industry

There was evidence of efforts to collectively create a coalition between government and industry to address the emergent issues around the pandemic collectively. This group consisted of representatives from all the impacted components of the waste sector together to discuss issues and create collective solutions. These issues captured, *"Will there be enough capacity to deal with waste? How to treat mixed waste containing medical waste? What were the risks to landfill operators?"* (P9)*.* There was also evidence from SA, where organisations have engaged strongly with the government, the industry and their customers. This has resulted in the organisation's preparedness and to be more resilient in the market. However, two government representatives (P24, P27) mentioned that due to the government COVID-19 recovery plan in response to the COVID-19 situation, which includes bringing the infrastructure projects forward to support local industry, the market would be more flourishing for the C&D waste stream after COVID-19. At the industry level, several interviewees stated that during COVID-19, the industry and government worked well together in response to issues that come with the pandemic. One interviewee had participated in several industry leadership calls with various state public organisations to communicate issues the industry faced during the affected period. The industry itself was quick in taking preventative containment measures. For example, *ʺthat's what I think was interesting, I mean you look at it and you thought, and this was in normal circumstances, the entire industry would say they need three years to implement these types of changes and they happened in three weeks. So, for me that was a really interesting phenomenon that we saw right across the industryʺ* [P11].

#### Diversifying supply chains and reducing supply chain risks

Diversifying the supply chain and creating more local transparent supply chains were suggested as key measures to mitigate risks. One participant stated that as their business operations in China provided them with some early opportunities to learn and navigate through disruptions, they could be ahead of the curve [P6]. There were early efforts to set up team-based systems, understand their procurement needs and mitigate the risks. Another participant reiterated the importance of reducing risks by creating secondary markets for C&D waste and enabling local supply chains. For example, "*I think any country should be looking at ways to reduce their exposure to supply chain risk, and if you can keep things onshore, that does a variety of things. It reduces supply chain risks, it creates jobs, and it creates alternative industry*" [P5].

Table 2 synthesises a range of responses to the COVID-19 impacts shared by interview participants across the five states of New South Wales (NSW), Queensland (QLD), Victoria (VIC), Western Australia (WA) and South Australia (SA).

**Table 2:** Specific responses to the COVID-19 impacts across five Australian jurisdictions

State	Key responses
NSW	Managing workforces with clearly defined shifts and cleaning arrangements
	Rapid interactions and tasks focused on prestart checks and cleaning
	Procuring masks and hand sanitisers
	Putting contingency and response plans in place very early and had communication out to the sites
	Creating clear communication channels and formulating clear messages
QLD	Increased attempts to use local products, diversify the supply chain as much as possible to reduce supply chain risks
	Leveraging virtual platforms to engage with other colleagues
	Creating a circular economy system via local markets and bringing the waste closer and the end products closer
	Requesting possible government funding for the industry to become profitable and to establish
	Conducting market assessments to assess the feasibility and to establish in the market
	Implementing COVID safety protocols
	Focusing on safe ways to work around waste, deliver essential services in local areas and across borders
	Creating a group of representatives from all the impacted components of the waste sector together to discuss issues and create collective solutions
	Identifying opportunities related to online platforms and real-time data, and digital engineering is positive repercussion of this disruption
VIC	At an individual level, measures such as social distancing, personal hygiene, taking care of one another, reporting if there is ever an issue with people having symptoms
	Repeating the message of the required practices and measures due to C&D waste recycling employees' unique socio-economic background
	Placing controls around shared spaces, and customer interaction
	Allocation of additional staff to each project called COVID monitors
WA	Developed targeted protocols in response to COVID-19 situations
	The extra construction work that has been undertaken is used up to stock balls or had some of those raw materials being turned into, such as recycled blocks or recycled road-based
	Creating a memorandum of understanding to share sources depending on the situation
	Release of planning caveats around the COVID-19 period to stimulate the economy
	Application of the segregation and the work-at-home requirements advised during the lockdowns
	Followed the advice from the state health authorities, such as social distancing, putting on a face mask
	The government's response was to try and fast track infrastructure projects as part of the economic recovery, which was an excellent opportunity for the government to increase the use of recycled C&D
	Working from home and preparing for the booming residential market
	The government responded to the COVID-19 situation by supporting the use of recycled content and increase in the number of projects that which has broadened the opportunities
**SA**	The industry and government worked together to address COVID-related issues
	The organisation implemented a 30% pay cut across their businesses to achieve this goal instead of laying off their employee

## Discussion

### Emergent impact matrix

As outlined in Results section, COVID-19 impacts on the industry have been diverse in the studied states. The following table provides an impact matrix developed based on interviewee responses. The impacts are categorised under five scopes: “construction and waste generation”, “employee engagement”, “transportation”, “processing and recycling” and “disposal”.

Table 3 shows that the construction and waste generation impact category have the most coverage in the interviews, followed by employee engagement. Furthermore, only “urgent implementation of safety protocols” was reported to impact all study states. Interestingly, restricted production and decline in demolition activities did not occur during pandemic restrictions. This is mainly related to the State COVID-19 policies, which consider construction and recycling essential industries. Such a matrix can be also used in other waste streams such as commercial and industrial waste (Aydin et al. 2017) and municipal solid waste (Abdallah et al. 2020) to investigate the potential impacts of the pandemic. Going forward, the authors summarise the following adaptive measures based on the findings.

**Table 3:** Summary of COVID impact matrix in five studied states

Scope	Impact description	States				
		NSW	QLD	VIC	SA	WA
Construction and waste generation	Interruptions to regular business operations	•	•	•		•
	Urgent implementation of safety protocols	•	•	•	•	•
	The decline of waste streams/limited material flows	•	•			•
	Interruption to supply chain	•		•		
	The decline in demolition activities	•				•
	Economic impacts/budget constraints					•
	Changing of priorities			•		•
	Increased infrastructure development due to government interjections			•		•
	Reduced tenders for construction			•		
	Delays in project planning and delivery			•		•
Employee engagement	Immediate need for remote working arrangements		•			
	Limited onsite engagement		•			
	Pandemic-induced human resource challenges (layoffs, pay cuts, increased workloads)			•	•	
Transportation	Restricted travel		•			
	Waste transportation and handling risks					
	Increased efficiency due to clear roads	•	•			
Processing and recycling	Restricted production					
	The decline in demolition activities/limited material flow					
Disposal	Stockpiling of materials unable to process		•			

### Adaptive measures

This section is concerned with objective 3, where the primary adaptive measures in place are to be explored. Based on the interviews and examination of relevant literature, the following measures are identified:

#### Government information support

State and territory governments have dedicated information hotline numbers for businesses affected by COVID-19. These information centres are currently operational in ACT, NSW, Vic, Qld, WA and NT. Notably, in Qld, the Chamber of Commerce & Industry Queensland has provided dedicated advisory services for small businesses. The Federal Government's business hotline also provides support for impacted small and medium enterprises.

#### Government financial support

State and territory governments have announced financial support for the businesses that are affected by the pandemic (Table 4). As expected, the highest level of financial support is offered by Qld ($2.5bn), NSW ($2.3bn) and Vic ($1.7bn) governments due to the size of their economy and population. Qld and ATC governments provide electricity rebates with a specific consumption threshold. All jurisdictions offer payroll tax relief in some capacity (Waste Management Review, 2020), between three and twelve months in duration. The federal government's financial support is done through three schemes, namely the Economic Recovery Package (ERP), Job Keeper Payment scheme (JKP) (Ananda 2020) and Tax-Free Cash Payment (TFCP). ERP provides support for the Australian economy, including small to medium enterprises, for three years. JKP aims to support employers to keep their staff; the eligible business should prove that their turnover was affected by COVID-19 by a fall of between 15 and 50%. TFCP provides tax-free payments between $20 k and $100 k. The government has committed more than $4.7 m to provide small regional businesses affected by COVID-19 with access to free and confidential financial counselling. In NSW and Qld, affected small businesses can apply for a re-opening grant (a one-off $3,000) and COVID-19 adaption grants ($10 k).

**Table 4:** The selected interview questions used in this study

Theme	Questions
Experience	Could you please introduce yourself and describe your role in your organisation? How long have you been involved in C&D waste management?
COVID-19 & waste management	In terms of the development of a market for recycled C&D waste products, please share your views on the conditions after COVID-19
	How has COVID-19 affected your organisation/industry?
	How does the industry respond to COVID during and after the outbreak?

The majority of responses to the ACOR COVID-19 Industry Pulse Check survey suggest landfill levy relief for 6 to 12 months [P16]. Other proposals include local governments waiving business fees; state and local governments becoming more flexible on certain facility license conditions for social distancing, so the protection of staff can be maintained; a landfill levy waiver on contaminated residuals from recycling facilities; payroll tax reductions; deferring business loan repayments; lifting collection time curfews so that bins can continue to be collected; and further support for tax and super obligations.

#### Interstate waste movement

The interstate waste movement has been primarily affected by restrictions imposed by Australian jurisdictions. NSW, Qld, Tas, SA, WA and NT have imposed movement restrictions across borders, but there are exemptions for essential travel, workforce movements, freight and specific compassionate grounds. For instance, NSW Public Health (COVID-19 Border Control) Order 2020 permits critical services, including construction and the maintenance and repair of critical infrastructure. The NSW Environmental Protection Authority (EPA) information fact sheet (April 2020) specified that the authority does not impose restrictions on interstate transport of waste. In NT and Tas, interstate travellers to NT must complete 14 days of self-quarantine unless they meet these exemption criteria. Travellers from declared hotspots to Qld are also required to complete 14 days of quarantine in government arranged accommodation (at their own cost) unless they are exempted from the requirements. In SA, while the board restrictions are ceased for travellers from WA, Tas and NT, travellers from other states are subject to an assessment of the public health risk. In WA, restrictions are in place, which disallows people to enter the state without an exemption.

### Implication for building circular built environment

This research has a policy implication for defining recommendations for circular economy as stated below. As envisaged by MacArthur (2013) and reinforced by other scholars (Della Torre et al. 2020; Munaro et al. 2020), one of circular economy objectives is to enhance resilience in the built environment sector. COVID-19 and associated uncertainties and impacts are an excellent opportunity to study potential pitfalls, impact areas, risk management, preventative strategies, and how stakeholders are adjusting to new conditions in both individual and collective manners. Future planning and practises that take into account all of these aspects will aid in the development of a resilient and circular built environment that will be prepared to accommodate uncertainties imposed by external factors such as pandemics.

This study by identifying the impacts of and responses to such uncertainties in the Australian context could inspire future circular economy guidelines. This fact also was echoed in the interview responses as presented in Table 2. Some of participants indicative statements collected in this study are provided below:

‘…there's obviously a long way to go, both in terms of C&D, and in terms of the waste industry itself. Re-evaluating where it sits and repositioning itself to be the frontend to the circular economy, and again …is an essential part of that’.

‘That's an example I think of putting things into place to head toward a circular economy, giving people the opportunity to consider non-virgin materials as well, as part of that exercise’ [P. 13].

‘I do believe that circular economy has actually come even more into focus and maybe has become something that people understand more than ever, just because of COVID. So circular economy really has to do with creating local markets and bringing the waste closer to yourself and the end products closer to where you live, and how you transport yourself and all of these things’ [P. 7].

‘I think there’s an opportunity there beyond C&D, and it sort of gets back to my point around the whole circular economy element that we need to think in the 20 year Waste Strategy, that we are going to start seeing more circulatory thinking, which sees materials coming in and out of C&I and C&D, moving through a broader life cycle process for materials, I think is gonna be really key’ [P. 3].

## Conclusions

The global influence of COVID-19 has impacted several sectors and businesses. However, there are few academic publications detailing these effects, particularly in the built environment sector. Consequently, the purpose of this research was to investigate these implications and explore how the business and government responded accordingly in the Australian context. This study adds to the body of knowledge regarding the impacts of COVID-19 on the C&D waste recovery industry in Australia. The interviews have shown that COVID-19 affected all industries in terms of work disruption, extra health & safety implications and impacts on the people involved. There are some specific impacts on the C&D waste industry in terms of the continued drive for homebuilding and supply chain issues. Impacts on the levels of recycling are mixed. In some instances, the sector reverted to unsustainable landfill and incineration, while in others, the COVID-19 impacts encouraged an increase in recycling practices. While previous research focussed on the economic impacts (Bell et al. 2022) and environmental impacts (Boroujeni et al. 2021) of COVID-19 on specific states in Australia, this research provides a more comprehensive understanding of a range of COVID-19 impacts on C&D waste management sector across five states in Australia.

A key study contribution is the emergent impact matrix developed from interviewee responses (Table 3). This matrix can facilitate risk management of uncertain conditions in five key areas: construction and waste generation, employee engagement, waste transportation, processing and disposal. Notably, construction (including waste creation), and waste recovery (including recycling) representing waste generation and consumption sectors, should be the focus of efforts to restructure existing policies and establish new ones. These two fields are also a significant source of employment (1 million people) (Kelly 2022), contribution to the country’ economy GDP (third in the economy) (Australian Industry and Skills Committee 2022) and solid waste management (first in solid waste creation and recovery) (National Waste Report 2020).

In terms of policy implications, this study provides the following four policy recommendations to further assist the industry with sustainable operation during these challenging times and provide a further contribution. Firstly, consideration of the C&D waste industry as an essential service in all Australian jurisdictions. Secondly, provide landfill levy relief for the next 6 to 12 months to stop illegal dumping and stockpiling. Thirdly, provide a landfill levy waiver on recycling residuals, particularly those which are contaminated. Fourthly, lift collection time curfews so that bins can continue to be collected.

The interviews have also demonstrated that in response to disruptions, such as COVID-19, the C&D industry needs to embrace digital and technological developments. While the study capitalises on the interviewee's knowledge and extensive industry experience, there is a limitation concerning the timing of the interview information gleaned concerning a fast-moving and evolving pandemic. The full impacts of the COVID-19 pandemic are yet to be known, and the findings from this study may become super-ceded by more important outcomes later on. However, these interviews did generate some novel and interesting insights, which includes lessons for other contexts. Hence, it is proposed that further studies investigate how the proposed adaptive measures from this study could be practically implemented and enable a resilient waste recovery industry.

A starting point for development of these measures is a set of practical government and industry tools from this study which could be implemented immediately and is listed in Table 5.

**Table 5:** A set of practical C&D waste management policy tools for government and industry

Sector	Policy tool
Government	Provide timely support for business through dedicated advisory services
	Offer financial support to businesses affected by Covid-19 pandemic through payroll tax relief, economic recovery grants and job keeper schemes
	Encourage local waste recovery through improved infrastructure
Industry	Use real-time data, digital engineering and other online technologies for effective communication, data management and maintenance tracking
	Develop business contingency plans for business continuation
	Diversify the supply chain to reduce risks and dependencies

## Data Availability

Enquiries about data availability should be directed to the authors.

## Footnotes

1 a payment to help keep Australians in jobs and supported businesses affected by the significant economic impact of the COVID-19.
